# Deconstructing Williamsburg: Using focus groups to examine residents' perceptions of the building of a walkable community

**DOI:** 10.1186/1479-5868-7-50

**Published:** 2010-05-27

**Authors:** Andrew T Kaczynski, Michael T Sharratt

**Affiliations:** 1 Department of Kinesiology, Physical Activity and Public Health Laboratory, Kansas State University, 1A Natatorium, Manhattan, KS, 66506, USA; 2 Research Institute for Aging, Faculty of Applied Health Sciences, University of Waterloo, Waterloo, Ontario, N2L 3G1, Canada

## Abstract

**Background:**

Components of the built environment are associated with active living behaviors, but research in this area has employed surveys and other quantitative methods almost exclusively. Qualitative approaches can provide additional detail about how neighborhoods influence physical activity, including informing the extent to which such relationships are causal in nature. The purpose of this study was to gain an in-depth understanding of residents' attitudinal and behavioral responses to living in a neighborhood designed to be walkable.

**Methods:**

Focus groups were conducted with residents of a planned retail and residential development that was designed to embody many attributes of walkability and was located within a large city in southwestern Ontario. In total, 31 participants provided qualitative data about neighborhood resources and dynamics, use of local services, physical activity behavior, and other related issues. The data were transcribed and coded for themes relevant to the study purpose.

**Results:**

Salient themes that emerged emphasized the importance of land use diversity, safety, parks and trails, aesthetics, and a sense of community, with the latter theme cutting across all others. The data also revealed mechanisms that explain relationships between the built environment and behavior and how sidewalks in the neighborhood facilitated diverse health behaviors and outcomes. Finally, residents recited several examples of changes in behavior, both positive and negative, since moving to their current neighborhood.

**Conclusions:**

The results of this study confirmed and expanded upon current knowledge about built and social environment influences on physical activity and health. That many residents reported changes in their behaviors since moving to the neighborhood permitted tentative inferences about the causal impact of built and social environments. Future research should exploit diverse methods to more fully understand how neighborhood contexts influence active living.

## Background

Improving physical activity levels has been identified worldwide as a top public health priority [1-5]. Physical activity contributes to a reduced risk of numerous chronic diseases such as obesity, cancer, osteoporosis, diabetes, cardiovascular disease, and depression [6,7]. Further, a lack of exercise is among the top three modifiable risk factors for premature death [8]. Economically, in 1995 dollars, the direct costs of inactivity accounted for $24 billion or 2.4% of U.S. health care expenditures, while costs associated with obesity accounted for an additional $70 billion [9]. In Canada, in 2001, about $5.3 billion of direct and indirect costs or 2.6% of total health care costs were attributable to physical inactivity, while obesity-related costs were estimated at $4.3 billion or 2.2% of total health expenditures [10]. Clearly, significant personal and societal costs are associated with physical inactivity and population-level strategies are necessary to improve upon these alarming statistics.

Modifying the built environment has been identified as one of the most promising strategies for increasing physical activity on a significant scale because of the relatively permanent effects of such changes and the ability to impact a large number of people [11]. Indeed, a growing and convincing body of evidence indicates that many neighborhood attributes are positively associated with active living behaviors. For example, reviews of this literature have reported fairly consistent connections between physical activity and factors in the built environment such as access to facilities, safety, and aesthetics [12-16]. Such reviews have also shown that transportation and planning variables such as mixed land use, population density, connectivity of streets, and the presence of sidewalks exhibit strong relationships with residents' physical activity levels [17-19].

Although research in this area has expanded exponentially in recent years, important limitations remain in the methods used to understand the impact of the built environment on physical activity. Generally, although some authors have used qualitative methods to good effect [20-22], quantitative approaches have been employed almost exclusively. Consequently, much is known about broad-scale associations between a wide array of environmental constructs and physical activity behavior [17]. However, these approaches generally provide little detail beyond what can be gleaned from the way scale items are worded or the way outcome data are contextualized. For example, concepts such as land use diversity are often measured by assessing distance to certain destinations, while less tangible constructs such as safety are often rated on a five-point scale [23]. Likewise, the impact of neighborhood attributes on behavior is inferred from activity counts on accelerometers or self-reported minutes on questionnaires, usually with little accompanying contextual information (e.g., where, with whom, experience of the activity, etc.). While advances in quantitative methods for measuring the built environment and activity-related outcomes are occurring [24-26], qualitative research about the role of the built environment in promoting physical activity is also needed.

Additionally, little research has examined the perspectives of residents who are living in walkable communities. Certainly, studies have documented differences in physical activity levels between people living in neighborhoods that are more or less endowed with features that support walking, including residential density, mixed land uses, greater connectivity of streets and trails, and abundant park space and recreational facilities [27-29]. However, as noted above, aside from these quantitative assessments, the viewpoints of persons experiencing these conditions in their daily lives have largely been ignored. Likewise, few studies have attempted to document the effects of a purposefully-designed walkable neighborhood (see quantitative research by Giles-Corti et al. [30] for an exception). Most studies to date have used cross-sectional designs, thus leaving it unclear as to whether walkable neighborhoods engender activity or whether active persons choose to live in walkable areas [17]. Although intervention or prospective studies (e.g., when a person changes residences) provide the strongest evidence of the impact of the built environment on physical activity, some researchers have integrated attitudinal questions into quantitative cross-sectional studies to demonstrate that built environments can affect walking behavior even after accounting for preferences for different neighborhood types [31,32]. However, qualitative methods can also offer insight about self-selection and causality by having participants directly describe connections between neighborhood features and their daily experiences and behaviors [33]. We explore such issues as one part of this paper. In summary, given the above considerations, the purpose of this study was to use an inductive approach to gain an in-depth understanding of residents' attitudinal and behavioral responses to living in a neighborhood designed to be 'walkable'. The study community and the focus group protocol used to explore such issues are described below.

## Methods

### Study Setting

This study focused on an area dubbed "Williamsburg" located on the outer edge of a large city (pop. 300,000) in southwestern Ontario. The neighborhood was not officially referred to as such but took its moniker from the adjacent commercial area called the Williamsburg Town Centre. Both the residential and commercial portions were part of a planned development by a local development company (owned, incidentally, by a former health studies professor). The study neighborhood was reasonably well-defined by two large arterial roads to the east and south, a higher volume connector road to the west, and a large plot of undeveloped land to the north. In total, the neighborhood comprised approximately 75 acres, including about 100 single-family and semi-detached homes and the commercial area which was about 12 acres in size and fronted one of the major roads. Because the neighborhood was not an official planning district or other census unit unto itself, it is difficult to estimate socio-demographic characteristics of the residents. However, based on the significant amount of time the authors spent in the neighborhood attending community events and visiting homes to solicit study participants, there appeared to be a substantial number of family-based households but also some older adult couples, and the majority of residents appeared to be from White, middle to upper income backgrounds.

At the time of the study (2007, approximately three years after development began), the retail section contained a hardware store (15,000 sq. ft.), a medium-sized grocery store (45,000 sq. ft), numerous (~20) additional smaller retail and service businesses (e.g., drug store, video store, dry cleaner, coffee shop, bank, salon, dental clinic, law firm), and some office space. Further, in addition to the commercial and residential sections, the neighborhood contained a central, well-maintained park area, approximately five acres in size containing several trees around its border, which had been set aside but not yet modified with any further landscaping or facilities. Three apartment buildings and five multi-residential villas had yet to be erected, but were planned for some of the vacant land closest to the shopping area and park.

From its initial planning, the Williamsburg Town Centre and broader neighborhood were developed with neo-traditional design principles in mind. The 13 guiding principles for the development, which were articulated over a decade prior to construction, included statements such as: "plan for mixed use that involves effective and integrated functioning among commercial, residential, and institutional uses", "maintain human scale in buildings and provide for diversity of architectural expression", and "encourage opportunity for meaningful human interaction within and beyond the community". Although the residential portion resembled an average suburban neighborhood of newer homes and few established trees, the streets were well-connected and contained sidewalks on both sides and all homes were located within easy walking distance of the commercial area. The Williamsburg Town Centre itself was functionally appealing to pedestrians; despite having a necessary parking lot in the middle, most of the buildings were composed of attractive red and yellow brick and adorned with window awnings, while sidewalks with old-fashioned light posts and trees permitted safe access on foot to most areas of the complex. Soon after it was developed, it attracted significant local media attention and informal accolades from city officials who were aiming to foster more pedestrian-scale development in newer areas of the city. As one reporter wrote, the Williamsburg Town Centre possesses "a distinctly retro-feel, something that hearkens back to the 1930s and 1940s before the automobile dominated most commercial design" [34]. Future plans included a 'main street' design closely fronted by the hardware store and other small shops on one side with additional stores with apartments above on the opposite side. The street, with traffic-calming roundabouts at either end, could be closed to traffic for community events and such an event occurred on a weekend during the time span of the present study. Overall, the neighborhood generally lived up to its slogan of "Your Village in the City".

### Data Collection

Guided by data collection and analysis methods described by Patton [35], this study employed an inductive approach to understanding residents' experiences living in this purposefully-designed walkable community. Residents' perspectives were gathered via focus groups that occurred in a meeting room at a business complex within the neighborhood. Focus groups were chosen because the topic under study was not deemed overly private or controversial, because the group format was more efficient than one-on-one interviews, and because of the potential for greater depth of findings to emerge as a result of the dynamic interplay of opinions among multiple community residents [36]. The minimal costs associated with the study were supported by the development company who were interested in residents' reactions to and experiences living in the neighborhood. However, the authors' more in-depth academic analysis of the data occurred independent of the services provided to the developer.

Given that the interest was on the experiences and opinions of current residents, a purposive sampling approach, based on location of residence, was employed. Specifically, participants were recruited via door-to-door distribution of flyers to all homes in the neighborhood during which a research assistant described the purpose and procedures of the study. Posters were also handed out and displayed at events around the community. Interested residents signed up for a convenient focus group time; no compensation was offered but light beverages and snacks were provided. During recruitment efforts, residents were told that the developer of the neighborhood was interested in residents' perspectives on what living in the neighborhood was like and how the experience might be improved. However, it was emphasized during recruitment and at the start of each group that the study was being conducted by independent researchers from the local university on behalf of the developer, that positive and negative feedback was welcomed, and that all responses would remain anonymous. At the start of each session, all participants provided informed consent of these parameters and of the fact that they could choose to avoid talking about any topic or could excuse themselves from the group at any point.

Each group lasted from 40 to 75 minutes and was structured around broad, open-ended questions about neighborhood resources and dynamics, use of local services, physical activity behavior, and related issues. Table 1 provides a list of questions which were used in the majority of groups, although additional topics and sub-topics were discussed as influenced by the conversation among the participants and focus group leader. The guiding questions were purposefully kept quite broad in order to allow participants' comments to drive the group discussions rather than directing the discussion toward preconceived themes from the conceptual or empirical literature. All groups were conducted in English and facilitated by the lead author. In total, seven focus groups were conducted in May through September 2007 involving 31 participants. Table 2 outlines selected socio-demographic characteristics of the participants as estimated (age, gender) or asked about (length of residence) by the focus group facilitator. Other characteristics (e.g., race, income) were not recorded or asked about, but the focus group participants appeared to largely represent the predominantly White, middle to upper income attributes of the neighborhood. During recruitment, no efforts were made to divide participants into groups according to age, gender, or other characteristics, so most groups contained a relatively heterogeneous mix of residents.

**Table 1:** Focus Group Question Guide.

Initial Questions	Follow-up Probes
Why did you choose this neighborhood?	Has the neighborhood met your expectations?
If someone asked you to describe your neighborhood, what would you say?	Why would you use those words?
Why did you choose this neighborhood?	Has the neighborhood met your expectations?
Is life different living here compared to in your previous neighborhood?	How? What makes it different or similar?
Are people physically active in and around the neighborhood?	What is it that encourages or discourages residents' physical activity?
Do you use Williamsburg Town Center?	Why? Which parts? How often? How do you get there?
What do you like most about living here?	Is there anything you don't like about living here?
What would you do to improve this neighborhood?	What are the most important priorities for change?

**Table 2:** Participant Characteristics.

Gender and Age	Length of residence in neighborhood			
	<1 year	1-2 years	>2 years	Total
Males				
18-34 years	1	3	2	6
35-54 years	0	2	1	3
>55 years	1	2	1	4
Total males	2	7	4	13
Females				
18-34 years	1	3	4	8
35-54 years	1	2	3	6
>55 years	1	2	1	4
Total females	3	7	8	18
Total	5	14	12	31

### Data Analysis

All focus groups were audio-taped after gaining consent to do so from the participants and were subsequently transcribed verbatim. Following procedures described by Patton [35], two researchers independently coded the raw transcripts for phrases and sentences that related to the study goals while developing relevant codes and themes that were shared and reviewed periodically. This iterative process continued until all transcripts had been reviewed several times and no new findings emerged, after which the final set of themes and representative data were agreed upon by both coders.

## Results

Participants in the focus groups discussed a range of topics related to positive and negative attributes of the neighborhood and the influence of these characteristics on their active and non-active behaviors. The following sections outline the key themes that emerged as coded and labeled by the researchers.

### Land Use Diversity

One of the most common types of comments centered around the convenience of having several different land uses within relatively close proximity to one another (e.g., residential, commercial, institutional, open space). This is frequently referred to as land use diversity or land use mix and is often considered a boon because it permits travel via active means between multiple destinations and home. Indeed, the mix of proximal land uses appeared to influence many residents' transportation choices, at least for those destinations that were accessible in the neighborhood. Some of their comments included:

*I work over at the [grocery store], so if I can, I walk to work ... it almost takes just as long to drive*.

I mean, everything's right there. You almost feel guilty if you drive!

Additionally, while most people described the benefits of mixed land use in terms of single-purpose excursions from residential to commercial areas (e.g., walking from home to the drug store), a few comments emphasized the convenience of having multiple retail and service businesses in one place:

*It's nice that you can walk over here from the neighborhood without having to cross a busy street and risk getting killed. And when you get here, you can take care of several things at once. It's not like [nearby box store shopping center] where you practically have to hop back in your car just to go to the next store. With the sidewalks, getting to and walking around the plaza is pretty easy, even for someone my age*.

*I like how I can check off a bunch of 'to-dos' in one trip ... groceries, prescription, bank, even get my nails done if I want! (laughs). Seriously though, it helps you save time and probably gas too*.

*It's nice to be able to get a lot of what you need in one place ... and when you're here doing your shopping for a while, you tend to bump into people you know*.

The benefits of this land use diversity were seemingly more salient for certain populations, as exemplified by the comments below from a mother with young children and an older adult:

*I don't want to move now. I can walk to the grocery store, I can walk to the post office. I have everything I need, which makes it easier as a parent with young kids. I prefer walking if I can as opposed to loading and unloading them from the car*.

*We're now in our retirement years and able to walk, so having the cleaners, [grocery store], hardware store so close ... it's having all these things together that makes it really a very nice place to live*.

Although most of the comments related to this theme focused on the commercial area in the neighborhood, several residents also appreciated the presence of the park space:

*It's cool having a park so close by too. We can just walk over with the dog and throw a ball around and then swing by the coffee shop on our way home*.

*Because the park is right there just up the road from my house, I think I feel like I should use it. You know what I mean? Might as well take advantage of it. There's not much to it right now, but I still like to walk the path around the perimeter in the morning*.

Finally, the very recent addition of an elementary (K-8) school to the community provided an additional dimension to the neighborhood's walkability. Numerous participants, almost exclusively those who were parents, reported changes in their behavior and that of their children as a result of the construction of the school:

*This year, our kids don't have to ride the bus and they can walk together to school and back*.

*Now, a lot of moms walk with their kids to school and then we all walk home together. You know, we're getting some exercise and also some social interaction*.

*Because there's a school close by now, our daughter's friends are more in the neighborhood now than when she went to [another school] which is way up [major artery street]. It's easier for us and she has more friends to play with on the weekend because they see each other during the week. The adults even know each other better too*.

Overall, land use diversity was the most common and strongly referenced theme throughout the groups. Residents generally had only positive comments about having of a variety of destinations within a short distance from home.

### Safety

Safety was another theme that emerged that tied together several of the comments from residents. Interestingly, participants provided slightly mixed reviews about neighborhood safety in that they felt quite safe from crime but had somewhat greater concerns about traffic issues. These diverse sentiments were exemplified in the following statements:

*We can trust leaving our kids outside ... we know most of the people who live around here and our street's not busy. The sidewalks help too so they don't have to play in the street*.

*I think it helps that they've tried to keep the neighborhood nice ... you know, with the nice architecture in the plaza and the streetlights and park and everything. It's a decent area so you're not too worried about anything bad happening*.

*Once in a while you get someone in a real hurry. But that's probably only on [major neighborhood road] out here. But I'm glad there's sidewalks or I wouldn't feel as comfortable walking anywhere*.

*It's terrible at times. It'd be nice to see them put in some sort of traffic calming measures on the busier streets*.

However, despite the mixed feelings, especially with respect to safety from traffic, there was general consensus that any safety concerns that did exist did not noticeably affect behavior:

*I don't think [the traffic] really prevents anything ... a lot of people are still out walking in the summer*.

*I'd say I feel pretty safe walking around the neighborhood ... even during rush hour when everyone's coming from work or at night time. There's sidewalks and lights and the people around here generally look out for each other*.

### Parks and Trails

As described above, residents valued the presence of a park in the neighborhood, even if, at the time of the study, it was largely an unimproved plot of open space (aside from regular mowing and some trees around the edge). Some also commented on other natural amenities they had discovered on the periphery of the community.

*It's nice to have that green space in the middle of the neighborhood, just to break up all the pavement*.

*The backyards in a lot of newer homes these days are pretty small so it's good to have a place where you can go just to throw a ball or let the kids run around*.

*That whole section of trees, there's nice little walking trails through there that I use with my dog ... when it's a hot, sunny day, it stays cool in there*.

However, despite the general appreciation for the park, some concerns were expressed about the space being underdeveloped and underutilized as a result:

*The field here - it'd be nice if it was something other than just a big open field. A playground would be ideal for parents with kids*.

*It would attract a sense of community, right? We'd actually come and hang out in one central location where you can house lots of neighbours*.

*Do you know what's happening with that space? Hopefully they put some stuff there to make it a bit more inviting*.

### Aesthetics

Fewer comments pertained to the idea of aesthetics, but several residents did seem pleasantly surprised at how appealing their neighborhood had become or remained:

*They've really maintained a lot of architectural control in designing the community*.

*The plaza is similar to what we had in the small town we came from, with awnings over the doors, nice brick ... the old-fashioned lampposts are a nice touch too. It's almost to the point where I actually enjoy shopping here ... like it's not a chore I have to get done. I mean, don't get me wrong, it's just a shopping centre, but they've at least put some thought into its design. And I think more people like coming here as a result*.

However, some residents expressed some frustration about the general 'newness' of the community:

*I miss the mature trees throughout the streets ... in older neighborhoods, there's more of a variety of home designs ... and less congestion*.

### Sense of Community

The final theme that emerged encompassed frequent comments, both complimentary and constructive, related to developing a sense of community. To begin, numerous residents expressed feeling a level of connectedness that they didn't necessarily experience in past neighborhoods in which they'd lived:

*There's a strong sense of community here ... you need a saw or a drill or something, you just go to your neighbor's house*.

*I think the fact that things are local, you're more apt to be out ... and having the park and plaza close by, people are more apt to be out because there are things to do*.

*People are generally pretty friendly. You know, you see them walking on the sidewalk past your house one day and you wave and they wave back. Next time, maybe they stop to talk*.

However, despite the numerous positive comments, several people expressed a desire and suggestions for facilitating increased community connectedness:

*The festival last weekend - was that really put on by the developers of the subdivision? - that was great. We actually got out and talked to people we sort of knew but had only waved at before. I hope they make it an annual event*.

*Some sort of community or neighborhood association would be helpful as this area grows*.

*A central message board where people can post events and other community notices might be useful ... We might chat with people out on the sidewalk during our evening walks, but otherwise we'd never know what was happening in the neighborhood*.

*This neighborhood is getting to a point now where we need - and deserve - a community centre or library; just something where you can go with your kids and see other families that's close by. Even a smaller common space in the new buildings that are going to be built would be a start*.

These and other comments about ideas related to community were some of the more passionate sentiments shared by residents during the focus groups. The importance of this notion is discussed further below.

### Behavioral Responses to a Neighborhood Change

The final theme that emerged was less centered around the influence of *specific *attributes of the built and social environments and instead addressed the broader notion of how a shift in one's overall habitat may influence behavior. Most of these comments came in response to a prompt inquiring about similarities and differences between participants' previous and current neighborhood and most responses focused on positive lifestyle changes that had occurred since the residents had moved to the Williamsburg area. For example, comments related to this theme included:

*Well, for one, as I mentioned earlier, we drive a lot less. It's nice having so many things within walking distance. In our old neighborhood, you had to use your car to get anywhere*.

*Now, there are definitely more things that are accessible that you might use on a day to day basis. It's funny because where we lived several years ago, we actually weren't too far from a small plaza with a grocery store, but you couldn't get to it unless you cut through your neighbor's backyard and crossed a busy street ... either that or walk way around the block and back down the next street over. So we always ended up driving there anyway*.

*Yes, I would say that living where we are now is quite a bit different than where we were before over in [neighborhood on opposite side of town]. We liked it over there and everything, but it didn't feel like we knew our neighbors as much. Now, we see people out walking, we run into them at [grocery store], we see them on the weekend when the kids are out playing. I'm not sure I could put my finger on why, but it definitely feels like there's more of a connection among the people living around here*.

*Oh yeah, it's totally different. Like our old neighborhood didn't have sidewalks and we were always scared to let the kids bike on the road. I mean, part of that was because they were younger then, but even right after we moved here, I remember feeling more comfortable letting them bike up and down the street, especially because there were other kids out doing so too*.

*You know, it's funny you should ask that because my husband and I were just saying the other day that there always seems to be a lot of people out walking around here. We just moved in this Spring and you can definitely see a difference versus where we lived before. And that goes for us too, especially on the weekend ... but even in the evenings some nights during the week, we'll go for a walk around the neighborhood or we'll walk up to get a coffee or to pick up some stuff for lunch the next day*.

Despite these positive sentiments, a minority of residents who relayed opinions related to this theme described less positive changes in their motivations and behaviors since moving to their new neighborhood:

*Yeah, it's certainly a bit different. There a lot of good things compared to the town we lived in previously, but we definitely miss some stuff too ... like here the neighborhood is still maturing and one of the big ways you notice that is with the lack of trees. There's no shade to keep the sun off you and it means we have to time when we take our walks which is somewhat annoying, especially for seniors who have all day to kill (laughs). My wife, especially, doesn't like to walk until the sun goes down because she complains about the heat ... at least in the summer ... and sometimes we're too tired by then which means we don't go at all*.

*You know, we generally like the neighborhood, but we can't wait until they do something more with the park. It's not exactly an exciting destination for the kids. In our old neighborhood, we were only about a 5-10 minute walk from [community park X] where there was a huge playground and we went there at least a couple times a week. Now that I think about it, I kind of miss that ... but hopefully we'll get back to doing that again soon if they put some stuff in the park here*.

A few other comments likewise conveyed negative behavior modifications as a result of the undeveloped park and a lack of trees, the primary frustrations described by participants in previous themes. However, the vast majority of lifestyle differences described by residents after moving to this largely walkable community were desirable. Regardless, both the positive and negative comments provide tentative evidence about how a neighborhood environment change can influence physical activity and other behaviors.

## Discussion

It is now relatively well-accepted that the contexts in which people live have significant implications for the ways they experience life events and for their overall health [37,38]. With this increasing recognition has come a concern for better understanding the factors in the built and social environments which are associated with health-enhancing behaviors such as physical activity [39]. Research on environmental influences on physical activity has proliferated in the past decade, thanks in no small part to advances in methodologies for both measuring neighborhood characteristics and for analyzing their associations with active behaviors. However, almost all environmental research has employed either quantitative surveys, observational audits, or data from geographic databases [24], while little attention has been paid to understanding the influence of neighborhood attributes through the descriptions of residents themselves. Thus, the purpose of this study was to use focus groups to explore how the building of a walkable community impacted the experiences and behaviors of the people therein.

### Discussion of Themes

The focus groups provided a wealth of information about a variety of different themes. Although greater detail about several topics was uncovered, many of the themes which emerged from participants' comments mirrored findings from quantitative studies about neighborhood and community factors that influence physical activity. For example, land use diversity, including the proximity of residential, commercial, institutional, and natural spaces, was mentioned frequently as a positive attribute of the community that affected residents' behaviors. Numerous studies of the built environment and physical activity have likewise reported that having a variety of destinations within walking distance is associated with increased amounts of walking and other activities [40-42]. Thus, the consistent findings across the two types of methods are mutually supportive. Residents also offered several comments about safety from traffic and crime, and survey and epidemiological studies have shown both perceived and actual safety to be related to levels of physical activity [43,44]. Moreover, a fair amount of research has documented positive associations between proximity to park space and physical activity [45-47], and the significance of the local park was another salient theme uncovered in our analysis. However, participants also expressed a desire for improvements to the park space in the neighborhood (e.g., playground), which is consistent with a growing body of research that shows that parks with more features are more likely to be used [45,48]. Finally, aesthetics were discussed as a key theme in this still-maturing neighborhood. Conceptualizations of aesthetics focused on notions of neighborhood greenness and architectural appeal or upkeep, all of which are ideas noted previously as relevant influences on physical activity [49,50].

In addition to corroborating relationships reported in the quantitative literature, our qualitative data also offered considerable insight into the mechanisms that may explain those associations. Kremers and colleagues [51] proposed a 'dual-process' model in which environmental factors can directly influence energy-balance behaviors (including physical activity) or in which the environment-behavior relationship is mediated by individual cognitions (e.g., attitudes, subjective norms, perceived behavioral control). The latter pathway seemed to be particularly prevalent in our study. For instance, some residents made comments related to feeling guilty about driving to destinations within the neighborhood or that they 'should' use particular amenities (e.g., the park) given how close they were to home. In this case, the increased diversity of proximal land uses seemed to create a normative imperative toward active choices. Future studies could explore how the design of walkable communities may create a culture that values and rewards - even expects - certain behaviors among residents. Likewise, other individuals conveyed how they cognitively weigh the pros and cons of decisions about where to go and how to get there. For example, a mother in one of our groups relayed how she contemplates the energy and time costs of loading and unloading children into the car versus walking to local destinations with them. Another parent described how his safety concerns about letting a child play outside were mitigated by environmental factors such as lower traffic volume and the presence of sidewalks. An older adult commented on the potential economic savings from driving less to accomplish everyday errands. Interestingly, few of the participants' comments about the benefits of the neighborhood were related explicitly to health outcomes or concerns about engaging in behaviors such as physical activity. Future research to promote the construction of walkable communities or to influence activity within them should explore the attitudes and outcomes that are most salient to residents' decisions (e.g., monetary cost, time, safety, community cohesion, health, etc.). Qualitative methods that build on the present study can help in elucidating the cognitive mechanisms by which environments influence behaviors.

Certain themes also emerged which provided new insights into the relationship between the built environment and physical activity. For instance, sidewalks were mentioned frequently throughout comments made about both the residential and commercial areas of the neighborhood. Because these comments often had a greater connection with another salient theme, they were not grouped together to form their own category. Nevertheless, the value of sidewalks was evident in the ways they served multiple functions in the community. For instance, various residents described the importance of sidewalks for getting to, from, and around the commercial area, while others mentioned their use of the sidewalk that encircled the neighborhood park. As well, despite several concerns about heavy traffic in certain areas of the neighborhood, the safety-related comments from some residents seemed to suggest that sidewalks provided a refuge for children's play and adults' recreational and utilitarian walking. Finally, sidewalks provided an informal meeting place for residents to interact and form connections with neighbors, potentially leading to the development of friendships and social capital. Some research has documented the association of sidewalks with physical activity behavior [52,53], but this study revealed a broader range of benefits as well as suggestions as to the mechanisms that may explain the link between the presence of sidewalks and increased neighborhood activity. Especially in light of research showing that sidewalk walkability can vary significantly across areas [54], future studies should continue to explore how investments in sidewalks can produce concrete outcomes related to multiple dimensions of health.

In addition to the individual themes described above, the collective comments of residents pointed to an overarching theme: the desire to build and experience community and how the design of the neighborhood facilitated or restricted such opportunities. This was discussed in the results section above as a category unto itself, but notions of community cut across almost all of the themes we constructed. For instance, participants discussed the proximity of multiple land uses or destinations as a factor that influenced their likelihood of interacting with neighbors on a regular basis (e.g., at the plaza, at the park). Likewise, the addition of the school to the community prompted greater interaction among both parents and kids during the week and on weekends. The sidewalks, as well, facilitated opportunities for passing communication between neighbors, while the park provided an important public space in this newer subdivision characterized by shrinking private lots. Finally, softer elements such as neighborhood aesthetics made the plaza and residential area more pleasant venues where relationships could be developed and sustained. At the same time, several enthusiastic comments related to a wish for even greater community cohesion as the neighborhood develops. To that end, residents offered numerous suggestions related to programmatic (e.g., events, neighborhood association) and structural (e.g., message board, community centre) changes that could be made to foster this increased connectedness.

At least some research has demonstrated how the built environment, in addition to promoting more physical activity, can affect issues related to community connectedness, neighborhood cohesion, and social capital. Indeed, proponents of new urbanism and smart growth philosophies adopt a broader vision of planning that emphasizes such goals for communities, thus aiming to make them not only more walkable but more 'liveable' and endowed with a greater sense of place. In one recent study, Cohen and colleagues [55] reported that certain neighborhood features, such as more parks and fewer alcohol outlets, were associated with individuals' ratings of collective efficacy (i.e., their perceptions of trust and of neighbors' willingness to help each other). In another study, Leyden [56] found that people living in more walkable neighborhoods, as measured by proximity to nine facilities or services, were more likely to know their neighbors, participate politically, have greater trust and faith in people, and be more socially engaged. Other research has reported that greater levels of social capital, community satisfaction, and community participation, among other indicators, are related to increased physical activity participation [57,58]. Overall, consistent with the findings of the present study, the design of neighborhoods and cities can significantly impact perceptions of 'community' among residents, which can have important implications for other health behaviors and outcomes as well. More research is needed to better understand the pathways between social capital and features such as land use diversity, density, connectivity, safety, and aesthetics, but this study provided some valuable evidence of the ways in which community was experienced and engendered in this walkable neighborhood.

Finally, an interesting theme emerged from our focus group data related to how residents experienced changes in behavior after moving to the Williamsburg neighborhood. Most participants described positive lifestyle changes related to less driving, more walking, and improved social support, but some also expressed how elements missing from their new neighborhood had negatively affected their physical activity patterns. The comments throughout this theme, both positive and negative, support the notion that environmental attributes do indeed have an impact on behaviors. The relatively nascent field of active living research, comprised almost exclusively of cross-sectional studies, has struggled for some time with the question of self-selection - whether activity-friendly neighborhoods promote greater physical activity or whether active persons seek out activity-friendly neighborhoods to reside in. Given the difficulty of changing most neighborhood features and the impracticality of randomly assigning people to live in designated areas, researchers have sought alternative methods for ascertaining whether a causal relationship exists between the built environment and physical activity and the extent to which self-selection is problematic in understanding such associations. Briefly, for example, Frank et al. [32] reported that participants often did not live in a walkable neighborhood, even if they preferred such an environment, and that even people with a preference for a low-walkable neighborhood walked more and drove less when living in a high-walkable area. In another study, Handy et al. [31] found that participants' perceptions of the built environment had an effect on walking behavior even after accounting for attitudes and preferences about different neighborhood types. By adopting such methods and measures in quantitative, cross-sectional studies, built environment and physical activity researchers have begun "stepping toward causation" [32]. However, the qualitative comments from residents in this study provided what is perhaps even more direct evidence of how a move from one environment to another can alter physical activity and other perceptions and behaviors.

### Study Strengths and Limitations

The qualitative approach adopted in this study proved useful in several ways. As expected, valuable detail was uncovered about concepts that past research has shown are significant environmental factors for physical activity. For example, participants' comments confirmed and elaborated on the importance of both safety from crime and safety from traffic and how elements of the neighborhood influence perceptions about these issues. Additionally, the focus group data not only confirmed past findings, but also provided novel insights into the ways certain neighborhood attributes help shape physical activity and other behaviors. Sidewalks, for instance, emerged as a key component of the neighborhood that not only permitted access around the residential and commercial areas, but also offered a venue for social interaction in both settings. Finally, having participants describe direct connections between neighborhood attributes and behaviors permitted tentative inferences about causality that extend the correlational findings which have dominated the built environment and physical activity research thus far. For example, the reactions of several participants to the building of a school in the neighborhood provided some retrospective evidence of the impact of a significant built environment modification that occurred right around the time of the study. In general, while there is certainly much merit in investigating these issues using a broader, perhaps more statistically representative sample, the qualitative methods used in this study provided additional depth about the attitudes and experiences of residents beyond that which could realistically be gleaned from surveys or other quantitative tools.

At the same time, the findings described herein should be interpreted in light of certain study limitations. For example, the dynamics of the focus group sessions could have skewed the data which were forthcoming. While the presence of other neighbors meant that participants were less likely to spout untruths and that they could build on each other's comments, there were points where the voices of certain residents dominated the conversation. However, attempts were made to minimize such occurrences and participants were continually encouraged to share divergent opinions. Another issue was that focus groups are inherently less flexible than solo interviews and, consequently, some interested residents were not available during the scheduled times (although groups were offered during the day and evening). It was also difficult to discern voices of individuals on the recordings, so the comments above are not attributed to residents of a particular age or length of residence. Another issue relates to the usefulness of gaining residents' perceptions in the first place. A latent debate exists in the built environment literature about the best way to measure environmental correlates of physical activity, and much research has shown that little correspondence exists between self-reported and objective measures of neighborhood features [59-62]. We felt that it was important to gather the subjective evaluations and experiences of residents, but objective measurements (e.g., audits, GIS) may be more appropriate for capturing certain community attributes. Similarly, participants' recall of life in a previous neighborhood versus the present one may deteriorate or become skewed, especially as time progresses. In addition, the study neighborhood and focus group participants lacked a significant amount of diversity with respect to race/ethnicity or socioeconomic status, so caution should be used in generalizing our findings to other settings. Finally, the authors acknowledge that the results of the study may be biased by any preconceived notions about the importance of the built environment in encouraging or discouraging physical activity.

## Conclusion

In summary, this study explored the perceptions of residents living in an apparently walkable community to better understand how attributes of their neighborhood influenced their feelings and behaviors related to physical activity and other health outcomes. In our experience studying this community, the focus group methodology proved very useful for capturing such information at a point when the neighborhood was beginning to take shape. Future researchers may wish to pursue a longitudinal study as such suburban communities evolve to examine, quantitatively and/or qualitatively, how changing neighborhoods of different designs impact the active and social behaviors of the people who live there. To combat the unrelenting crises of obesity [63] and social isolation [64], understanding, from all available viewpoints, how neighborhood and community features influence physical and mental health will become increasingly critical to public health research and practice.

## Competing interests

The authors declare that they have no competing interests.

## Authors' contributions

Both authors participated in conceptualizing the study. AK led all aspects of data analysis and writing and MS contributed to writing and editing. Both authors have read and approved the final manuscript.
